# Pericardial Tumor, a Rare Manifestation of Sjogren’s Syndrome Secondary to Systemic Lupus Erythematosus

**DOI:** 10.7759/cureus.11069

**Published:** 2020-10-20

**Authors:** Muhammad Sohaib Asghar, Urooj Shuja, Saira Anwar, Maira Hassan, Uzma Rasheed

**Affiliations:** 1 Internal Medicine, Dow University of Health Sciences, Karachi, PAK; 2 Internal Medicine, Liaquat National Hospital, Karachi, PAK

**Keywords:** ana, autoimmune, tumor, pericardium, syndrome, antibody, sjögren’s, ds-dna, sle, eular

## Abstract

We are presenting a case of pericardial tumor in an elderly female patient who presented with low-grade fever, purpuric rashes all over the body, grittiness in the eyes, and dry mouth with decreased oral intake, night sweats, weight loss, chest pain, and dyspnea. She was diagnosed with Sjögren’s syndrome secondary to systemic lupus erythematosus (SLE) with positive anti-nuclear antibody (ANA), anti-double-stranded DNA (anti-ds-DNA), and anti-Sjögren's-syndrome-related antigen A autoantibodies (SS-A/Ro) antibodies. Computerized tomography scan of the chest with contrast showed multiple calcified mediastinal lymph nodes and a well-defined solid cystic lesion adjacent to the left atrial appendage in favor of a pericardial tumor with minimal pericardial effusion. Biopsy could not be done due to the risk of cardiac tamponade and pneumothorax secondary sensitive location of the tumor. The patient was referred to the oncology and cardiothoracic surgery department for an opinion regarding resection of the tumor and further palliative management. This case is unique in a way that the current literature does not associate SLE with pericardial tumor, while our patient had no other primary malignancy or secondary metastasis ruled out on a positron emission tomography (PET) scan.

## Introduction

Pericardial tumors rarely originate from the pericardium or metastasize from other sites. The primary benign tumors include pericardial cysts and lipomas. Malignant neoplasms include sarcomas, lymphomas, primitive neuroendocrine tumors, and mesotheliomas, being the most common malignant pericardial neoplasm. The first case of pericardial mesothelioma with autoimmune features dates back to 1984 [1]. A total of 150 cases of primary mesothelioma of the pericardium have been reported to date [2]. They usually present as shortness of breath, chest pain, fever, weight loss, and palpitations. They can cause compression of vital mediastinal structures and permeate great blood vessels and myocardium and result in metastatic disease. The prognosis of benign tumors is good while that of malignant mesothelioma is bleak. Echocardiography is the preferred modality for initial evaluation. Computerized tomography (CT) scan provides superior spatial resolution and is the ideal test for calcified masses while cardiac magnetic resonance provides excellent tissue characterization. Positron emission tomography (PET) gives additional information on the assessment of potentially malignant tumors [3,4]. Surgical resection can be curative in early stages and for localized tumors. Pericardiectomy, chemotherapy, and radiotherapy are often used as palliative approaches [5].

Our report highlights a case of Sjögren’s syndrome secondary to systemic lupus erythematosus (SLE) in a patient who had long-term symptoms of suggestive autoimmune disease, with no family history of SLE or Sjögren's. The patient was started on topical fluorides, tear and saliva substitutes along with prednisolone (1 mg/kg) and hydroxychloroquine (200 mg, twice daily). A computerized tomography scan of the chest with contrast showed an incidental finding of a pericardial tumor.

## Case presentation

A 70-year-old female of Asian ethnicity, known case of hypertension, presented to us with complaints of fever, itchy purple-colored rashes all over the body, grittiness in the eyes, and dry mouth with decreased oral intake, vomiting, night sweats, weight loss, dyspnea, and chest pain. The patient also had recurrent oral ulcers and alopecia but there was no joint pain, morning stiffness photosensitivity, or Raynaud’s phenomenon.

**Figure 1:**
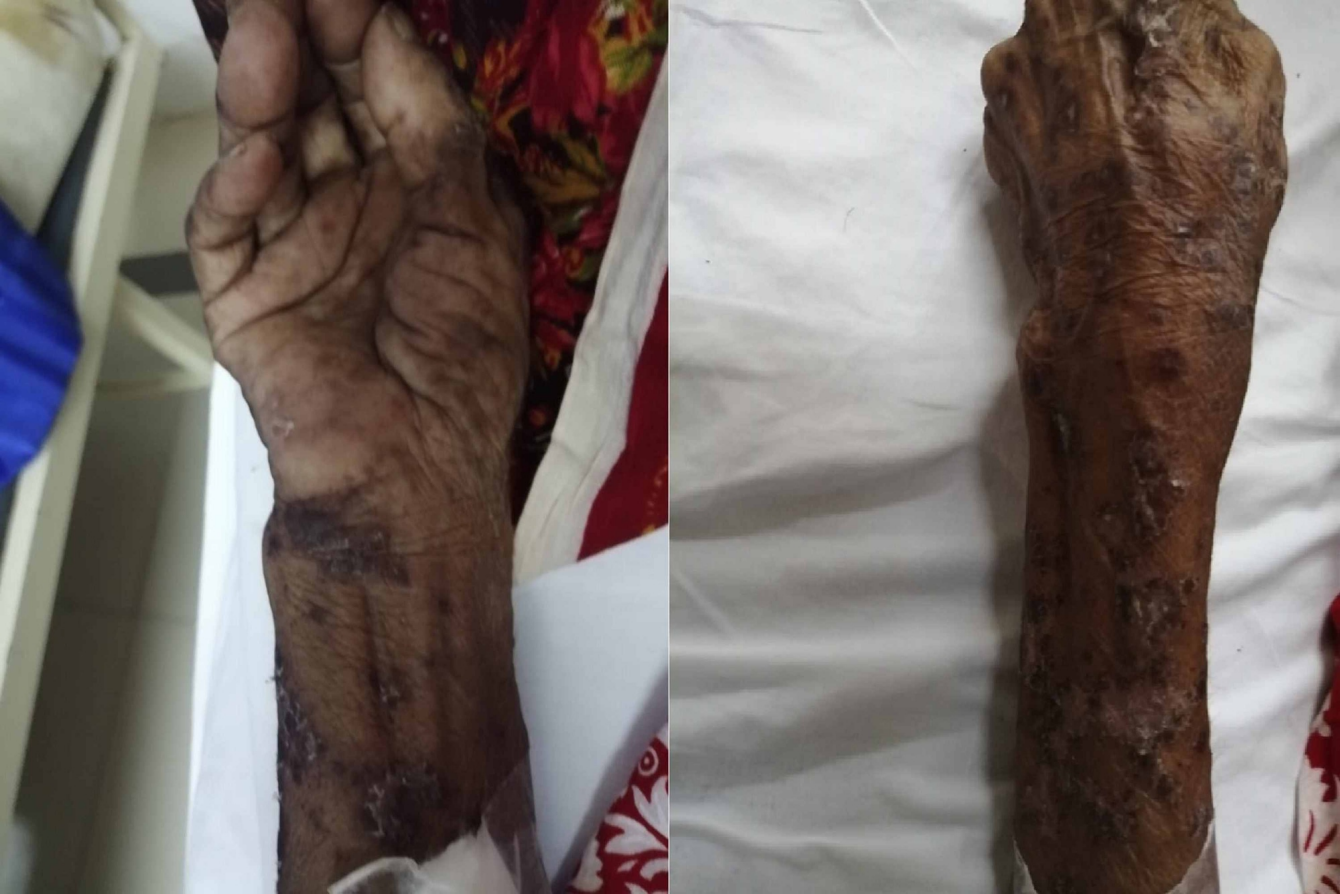
Non-blanchable, purpuric, ulcerated rashes on the upper limb.

On physical examination, thin build, pallor, poor skin turgor, koilonychia, angular cheilitis, oral ulcers, glossitis were the positive findings along with multiple palpable non-blanchable purpuric rashes on the limbs (Figure 1) and abdomen. Chest auscultation revealed inspiratory crackles on lower zones bilaterally with decreased vocal resonance in the left mid-zone. Initially, the patient was given intravenous antipyretics, antihistaminics, and oral nutritional supplements.

The laboratory findings of the patient are presented in Table 1.

**Table 1:** Baseline investigations and relevant autoimmune workup of the patient.

Laboratory investigation	Value	Reference range
Hemoglobin	8.8 mg/dL	12.0-15.0 mg/dL
Mean corpuscular volume	83 fL	80-100 fL
Total leukocyte count	4.8 cells/microliter	4.0-10.0 cells/microliter
Platelet count	250x10^9^ per microliter of blood	150-400 x10^9^ per microliter of blood
Erythrocyte sedimentation rate	102 mm/hour	<20 mm/hour
C-reactive protein	27 mg/L	<5 mg/L
Direct Coombs test	Positive (with 2+ titers)	Negative
Reticulocyte count	0.8%	0.5-1.5%
Serum albumin	2.16 g/dL	3.5-4.5 g/dL
Urine detailed report	+1 proteinuria	Negative
Antinuclear antibody (ANA)	Positive	Negative
Anti double-stranded DNA (Anti-dsDNA)	Positive	Negative
Anti Sjögren’s syndrome-related antigen A (Anti SS-A/Ro)	Positive	Negative

**Figure 2:**
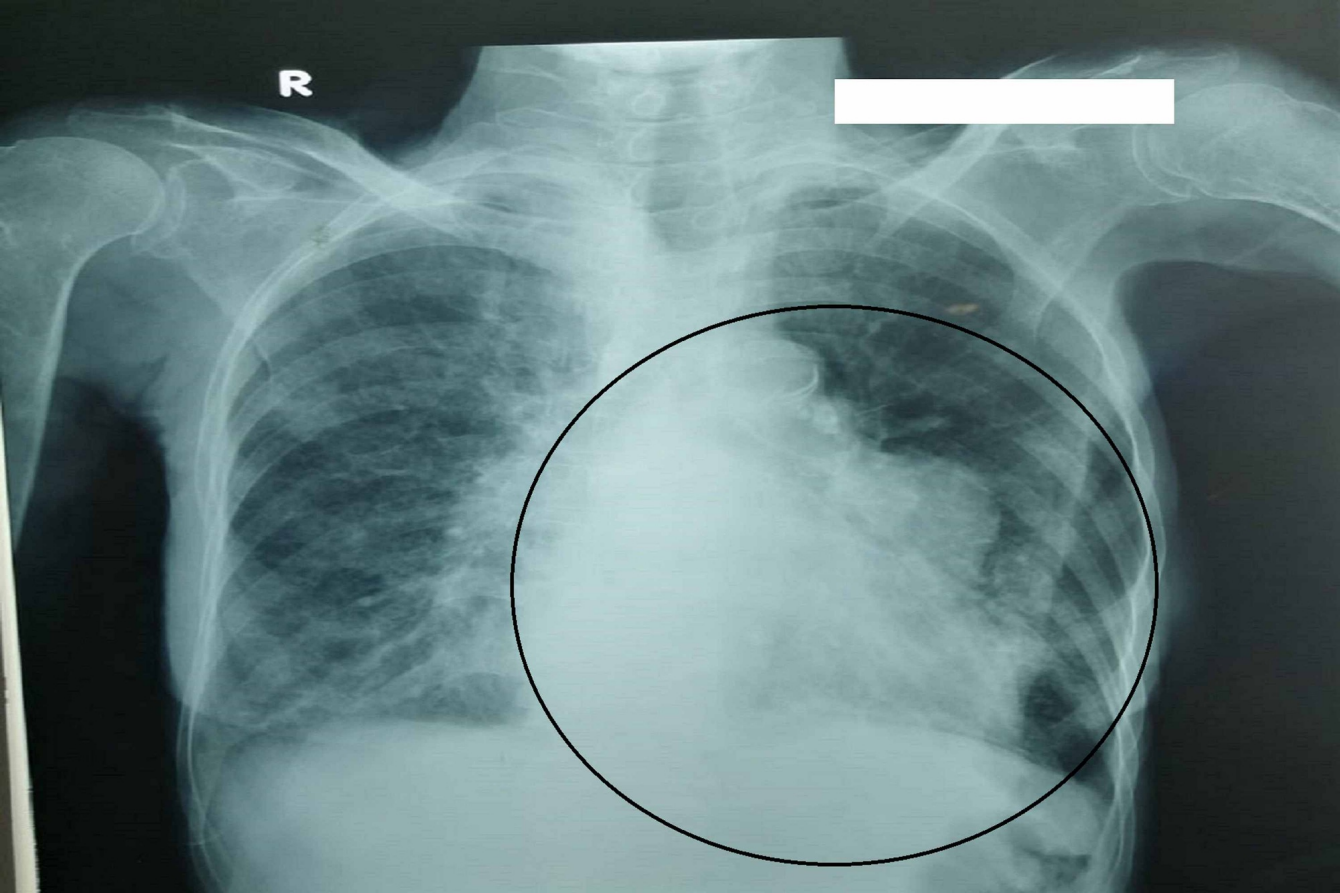
Chest X-ray showing cardiomegaly along with irregular left cardiac border mimicking homogenous opacity in left mid-zone of the lung.

The rest of the autoimmune workup was negative. A primitive diagnosis of Sjögren’s syndrome secondary to systemic lupus erythematosus was made and further evaluation of the patient’s dyspnea was planned to rule out interstitial lung pathology. Chest X-ray showed cardiomegaly (Figure 2), while transthoracic echocardiography showed minimal pericardial effusion.

**Figure 3:**
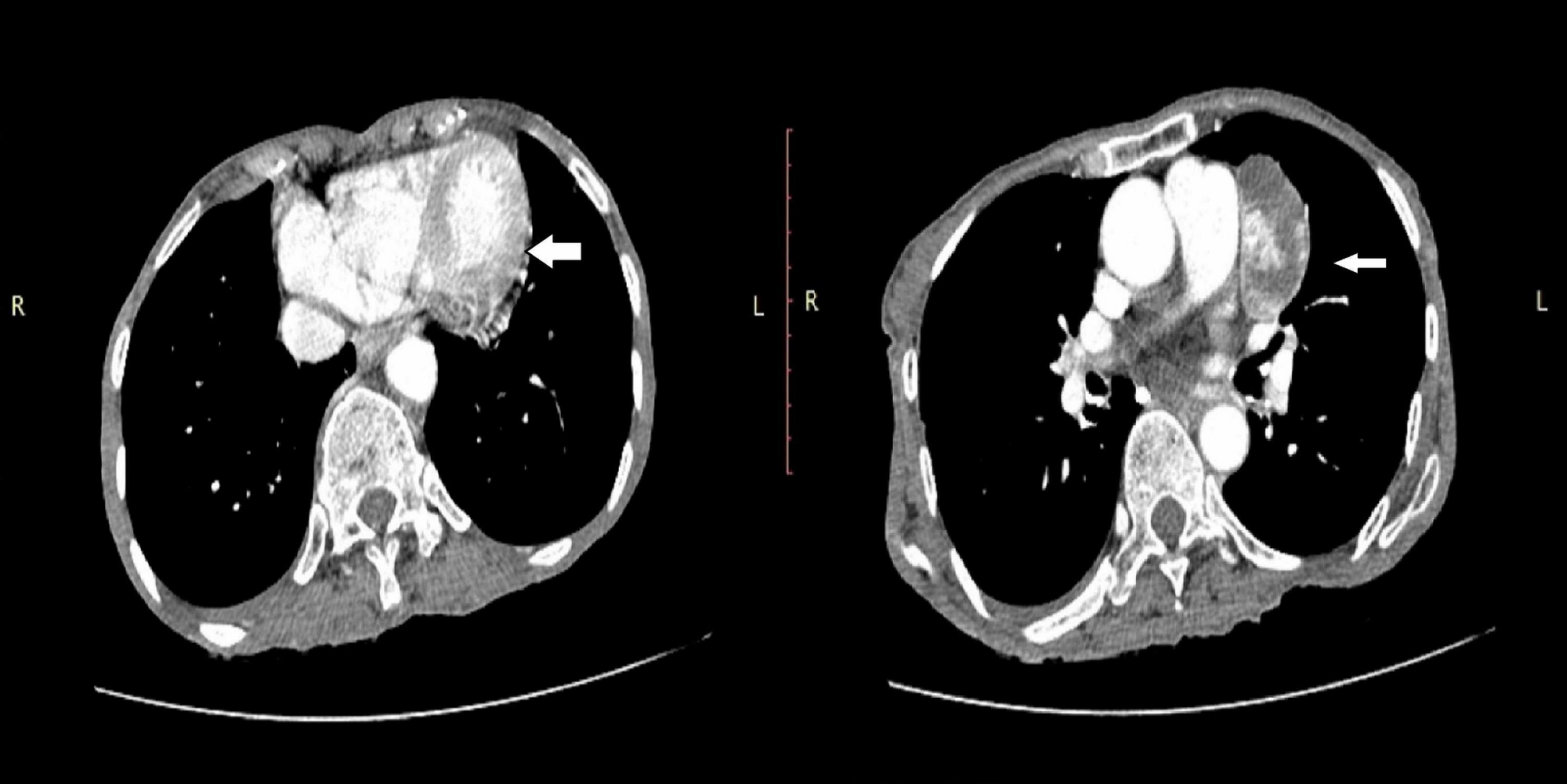
Computerized tomography scan of the chest with contrast (axial view) showing well-defined solid cystic lesion adjacent to the left atrial appendage inseparable from the pericardium.

**Figure 4:**
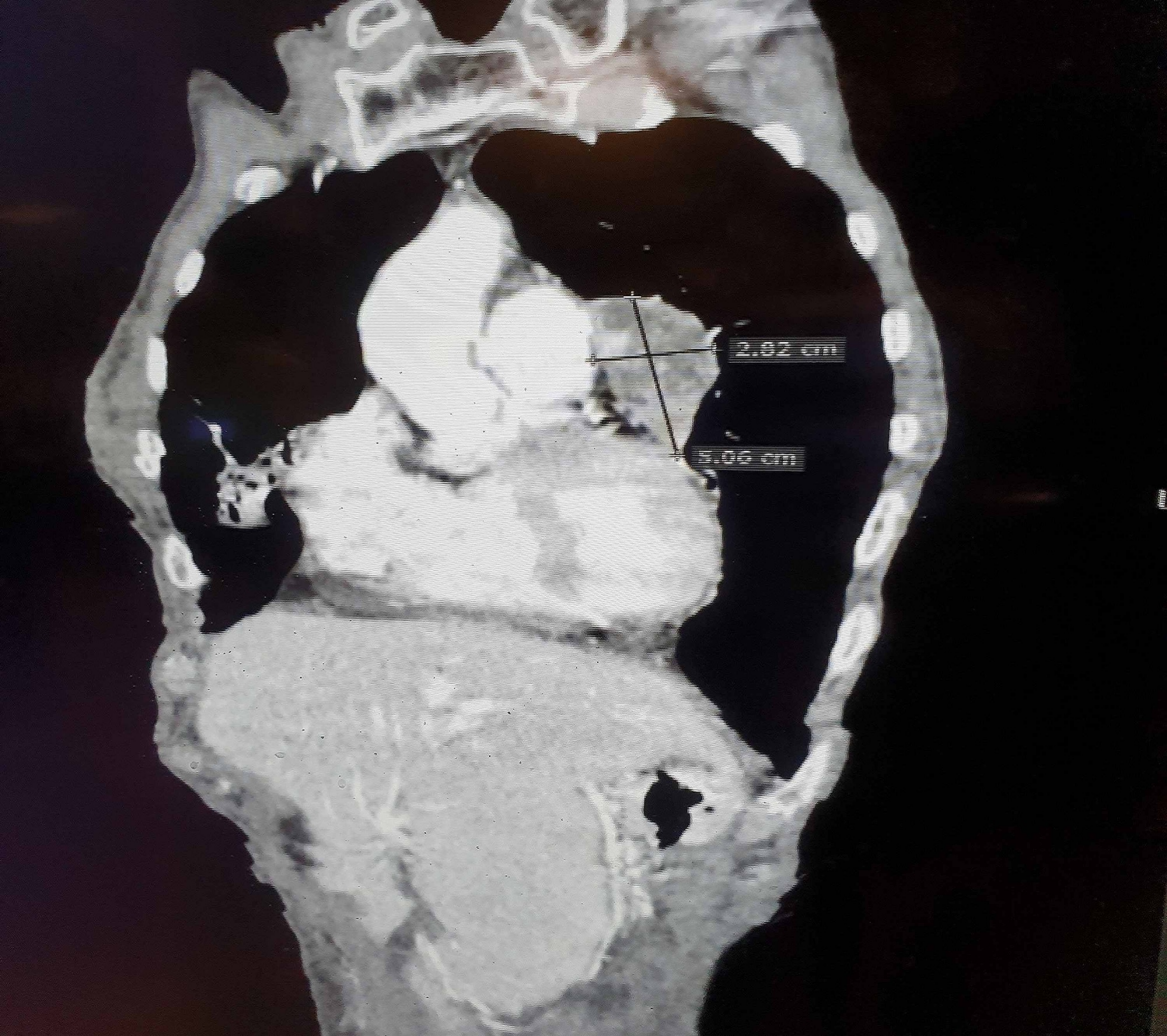
Computerized tomography scan of the chest (coronal view) showing pericardial tumor of 5.06x2.82 cm along with some mediastinal lymph nodes.

Computerized tomography scan of the chest with contrast was carried out, which showed an incidental finding of cardiomegaly with a well-defined solid cystic lesion of 5.06x2.82 cm adjacent to the left atrial appendage inseparable from the pericardium, in favor of a pericardial tumor along with some mediastinal lymph nodes (Figure 3, 4). Differential diagnoses considered were sarcoidosis, amyloidosis, tuberculosis, and mediastinal lymphoma. PET scan was carried to rule out any primary malignancy or secondary metastasis, which came normal. The patient was diagnosed as a case of Sjögren’s syndrome secondary to systemic lupus erythematosus and was started on prednisolone (1 mg/kg) and hydroxychloroquine (200 mg, twice daily) for an initial two months. Biopsy could not be done due to the sensitive location of the tumor and the risk of cardiac tamponade and pneumothorax. It is also important here to mention that the patient was not willing for any invasive procedure, hence she was referred to the oncology department for further palliative management. The patient’s clinical course could not be followed because she was lost to follow-up.

## Discussion

The term Sjögren’s syndrome was first described by Swedish ophthalmologist Henrik Sjögren who linked the triad of keratoconjunctivitis sicca, xerostomia, and polyarthritis. The first reported case of dry eyes and dry mouth was elucidated by Hadden and Hutchinson in 1871 [6]. Primary Sjögren’s syndrome is very rare with a worldwide prevalence of 61 per 100,000 individuals, being highest in Europe and overall prevalence including secondary Sjögren is 0.4% [7]. Sjögren's syndrome is a systemic autoimmune disorder causing lymphocytic infiltration of the salivary and the lacrimal glands that leads to fibrosis and exocrine failure. It can be primary or secondary to other autoimmune diseases like systemic lupus erythematosus, rheumatoid arthritis, systemic sclerosis, celiac disease, primary biliary cirrhosis, myasthenia gravis, and chronic active hepatitis. It is associated with HLA-B8/DR3 [7]. The typical age of onset is 40 to 50 years with a 9:1 ratio between females and males and predominantly affects Caucasians. Secondary Sjögren’s syndrome is most commonly associated with systemic lupus erythematosus in 15 to 36%, rheumatoid arthritis in 20 to 32%, limited or diffuse systemic sclerosis in 11 to 24% of the cases, and also with granulomatosis with polyangiitis and polyarteritis nodosa. There is a 5% risk of non-Hodgkin’s lymphoma [7], and there is a 4% risk of lymphomas involving salivary glands, stomach, lungs, and adrenal glands. There is scarce data available for cardiac lymphoma usually in individuals who were human immunodeficiency virus-positive, 21.2% had pericardial effusion evident [8].

The common clinical features of Sjögren’s syndrome include keratoconjunctivitis sicca, xerostomia, salivary gland enlargement, rashes or skin irritation, vaginal dryness with dyspareunia, non-erosive arthralgias, generalized osteoarthritis, Raynaud’s phenomenon, and fatigue, while less common features include low-grade fever, interstitial lung disease, anemia, leucopenia, thrombocytopenia, vasculitis, cryoglobulinemia, lymphadenopathy, peripheral neuropathy, glomerulonephritis, interstitial nephritis, and renal tubular acidosis [9]. Keratoconjunctivitis sicca is characterized by dryness of eyes due to the inflammation of the lacrimal gland either due to decreased production of tears or increased evaporation of tears which can lead to keratitis and blindness [10]. According to the American College of Rheumatology/European League Against Rheumatism (ACR-EULAR) criteria [11], a score of ≥4 is diagnostic of Sjögren's syndrome with labial salivary gland biopsy showing lymphocytic infiltrates, positive anti-SSA/Ro antibodies, positive Schirmer test less than 5 mm in five minutes, and salivary flow rate less than 0.1 ml/min along with dryness of eyes or mouth. Patients with sarcoidosis, amyloidosis, acquired immunodeficiency syndrome, active hepatitis C infection, graft versus host disease, immunoglobulin G4-related disease (IgG4-RD), and radiotherapy to the head or neck are excluded from the criteria [11]. Treatment options include tear and saliva substitutes, pilocarpine, cevimeline, cyclosporine and corticosteroid eye drops, and topical fluoride [12]. Poor prognostic factors showing reduced survival rates include purpura, low C4 complement levels, and mixed monoclonal cryoglobulinemia [9].

SLE can increase the risk of many malignancies including non-Hodgkin’s lymphoma, Hodgkin’s lymphoma, leukemia, multiple myeloma, cervix, vagina/vulva, kidney, bladder, esophagus, gastric, hepatobiliary, lung, oropharynx, larynx, non-melanoma skin, and thyroid cancers, while it could reduce the risk of prostate cancer, breast cancer, and cutaneous melanoma [13]. Additional imaging studies like magnetic resonance imaging and PET scan have conventionally helped in reaching a definite diagnosis before the biopsy. Additionally, it would also be useful to include the oncologist's and surgeon’s evaluations regarding the tumor resection as well as further management, which was not available in our case due to loss of follow-up. Pericardial involvement in autoimmune diseases is usually limited to pericarditis and pericardial effusion. Pericardial tumors are potentially rare, the most common benign lesions are pericardial cysts and lipomas. Mesothelioma is the most common malignant pericardial tumor usually associated with asbestos exposure. Other malignant tumors may include sarcomas, lymphomas, and primitive neuroectodermal tumors. One such case study presented mesothelioma of the pericardium with suggestive autoimmune features mimicking lupus [1].

## Conclusions

As our case presented with fever, grittiness in the eyes, dry mouth, dyspnea, recurrent oral ulcers, alopecia, and non-blanchable purpuric rash, a workup suggestive of autoimmune pathology was carried out and returned positive diagnosing Sjögren’s syndrome secondary to systemic lupus erythematosus. Although it is interesting to see both of these together in the same patient as a new-onset at such an advanced age, it is also a well-known fact that one autoimmune disorder pre-disposes to the others. Our report highlights a rare association of a pericardial tumor with an autoimmune entity. SLE is associated with many cancers, while there are few cases in literature associating Sjögren’s syndrome with mesotheliomas which can include pericardial tumors also. This case is unique in a way that the current literature does not associate SLE with pericardial tumor, while our patient had been ruled out for any other primary malignancy or secondary metastasis.
